# KPC-3-Producing Klebsiella pneumoniae Sequence Type 392 from a Dog’s Clinical Isolate in Portugal

**DOI:** 10.1128/spectrum.00893-22

**Published:** 2022-06-23

**Authors:** Joana Moreira da Silva, Juliana Menezes, Gabriel Mendes, Sofia Santos Costa, Cátia Caneiras, Laurent Poirel, Andreia J. Amaral, Constança Pomba

**Affiliations:** a Centre for Interdisciplinary Research in Animal Health (CIISA), Faculty of Veterinary Medicine, University of Lisbon, Lisbon, Portugal; b Associate Laboratory for Animal and Veterinary Sciences (AL4AnimalS), Lisbon, Portugal; c Microbiology Research Laboratory on Environmental Health (EnviHealthMicro Lab), Institute of Environmental Health, Faculty of Medicine (ISAMB), University of Lisbon, Lisbon, Portugal; d Global Health and Tropical Medicine (GHTM), Instituto de Higiene e Medicina Tropical (IHMT), Nova University of Lisbon, Lisbon, Portugal; e Institute of Preventive Medicine and Public Health, Faculty of Medicine, University of Lisbon, Lisbon, Portugal; f Medical and Molecular Microbiology Unit, Faculty of Science and Medicine, University of Fribourg, Fribourg, Switzerland; g Genevet, Veterinary Molecular Diagnostic Laboratory, Carnaxide, Portugal; University of L'Aquila

**Keywords:** Carbapenemases, companion animals, multidrug-resistant *Klebsiella pneumoniae*

## LETTER

Resistance to carbapenems in Enterobacterales poses a threat to health care systems worldwide since those infections are associated with high mortality and limited treatment options. The latest European reports show that some southern European countries are almost endemic for carbapenemase-producing Enterobacterales (CPEs) (1, 2). Mirroring the European tendency, Portugal has been observing a steady increase in the occurrence of carbapenem-resistant Klebsiella pneumoniae, particularly KPC-3-producing K. pneumoniae ST147 lineage (Clonal Group 147) strains in health care settings (3, 4).

To assess the relevance of CPEs in veterinary health care, 977 Enterobacterales isolates obtained at the veterinary molecular diagnostic laboratory (Genevet) during 2020 were screened for carbapenem resistance. Phenotypic disc testing with 10 μg meropenem, 30 μg temocillin, and MAST CAT-ID discs (Mast Group, UK) and genotypic confirmatory tests were performed to detect plasmid-mediated AmpC β-lactamase and ESBL- and carbapenemase-producing Enterobacterales (5, 6).

Interestingly, a carbapenem-resistant and carbapenemase-producing K. pneumoniae isolate (VG380) was recovered, being positive for the *bla*_KPC-type_ gene. It was recovered from a nasal swab collected from a 10-year-old dog with an upper respiratory tract infection. Cytology of the nasal exudate displayed an abundance of degenerated neutrophils, bacilli, and cocci. Biopsy of the nasal tissue displayed an ulceration showing signs of mixed inflammation, with an abundance of neutrophils, lymphocytes, macrophages, and dense fibrovascular proliferation, which was compatible with the diagnosis of chronic ulcerative rhinitis. A 1-month course of treatment with trimethoprim-sulfamethoxazole was followed and the dog made a full recovery.

K. pneumoniae strain VG380 was resistant to ertapenem, meropenem, and imipenem (MIC >1 mg/L for ertapenem and >8 mg/L for imipenem and meropenem; determined by MicroSan NEG44 plates [Beckman Coulter, USA]). It was susceptible to ceftazidime-avibactam (MIC = 2 mg/L; determined by Etest [bioMérieux, France]). Interpretation of MIC determinations followed EUCAST breakpoint tables (https://www.eucast.org/fileadmin/src/media/PDFs/EUCAST_files/Breakpoint_tables/v_12.0_Breakpoint_Tables.pdf).

Whole Genome Sequencing (WGS) analysis of strain VG380 was performed using Illumina NovaSeq platform with 2 × 150-bp paired-end reads. The quality of the resulting raw reads was evaluated using FastQC v0.11.5 (https://www.bioinformatics.babraham.ac.uk/projects/fastqc/). *De novo* genomes were assembled using SPAdes v3.14.1 (7), following two rounds of polishing with Pilon v1.24 (8). The generated assemblies were used to screen for antimicrobial resistance genes and mobile genetic elements resorting to tools available at Centre for Genomic Epidemiology (http://genomicepidemiology.org/), ResFinder 4.1 and Mobile Element Finder v1.0.3, respectively. MLST 2.0 and pMLST 2.0 were also performed.

WGS analysis showed that VG380 possessed a KPC-3-encoding gene and belonged to Sequence Type ST392, a single-locus variant of ST147, also belonging to Clonal Group 147. Therefore, this canine strain was related to the KPC-3-producing K. pneumoniae ST147 lineage responsible for the majority of nosocomial infections reported so far in Portugal (3, 4). Virulence factors *traT* and *iutA* were identified, with the former being located on a IncFII(K)-type plasmid. IncFIB(K)- and IncN- type plasmids were also identified. Noteworthy, strain VG380 harbored a series of resistance genes leading to a quite unusual multi-drug resistance profile for a canine isolate (Table 1).

**TABLE 1:** Antimicrobial MICs and resistance genes identified during WGS analysis on KPC-3- producing Klebsiella pneumoniae ST392^*a*^

Antimicrobials tested	MIC (mg/L)	Susceptibility phenotype^*b*^	AMR genes
Amikacin	≤8	S	*aac(6’)-lb-cr*
Ampicillin	>16	R	*bla*_TEM-1B;_ *bla*_SHV-11;_ *bla*_CTX-M-15;_ *bla*_KPC-3_
Amoxicillin-clavulanic acid	>16/8	R^*c*^	*bla*_CTX-M-15;_ *bla*_KPC-3_
Aztreonam	>16	R	*bla*_CTX-M-15;_ *bla*_KPC-3_
Cefotaxime	>32	R	*bla*_CTX-M-15;_ *bla*_KPC-3_
Ceftazidime	>16	R	*bla*_CTX-M-15;_ *bla*_KPC-3_
Ceftazidime-avibactam	2	S	NA
Ciprofloxacin	>2	R	*aac(6’)-lb-cr; OqxB/A; qnrB1*;
Colistin	≤2	S	NA
Ertapenem	>1	R	*bla*_KPC-3_
Gentamicin	≤2	S	*aac(6’)-lb-cr*
Imipenem	>8	R	*bla*_KPC-3_
Meropenem	>8	R	*bla*_KPC-3_
Tetracycline	>8	R^*c*^	*tet(A)*
Trimethoprim/sulfamethoxazole	≤2/73	S^*c*^	*sul2; OqxB/A*

a S, susceptible; R, resistant; NA, not applicable; AMR, antimicrobial resistance; WGS, whole genome sequencing.

b Susceptibility phenotype was determined according to EUCAST breakpoint guidelines.

c Susceptibility phenotype was determined according to Clinical and Laboratory Standards Institute guidelines (9).

**FIG 1:**
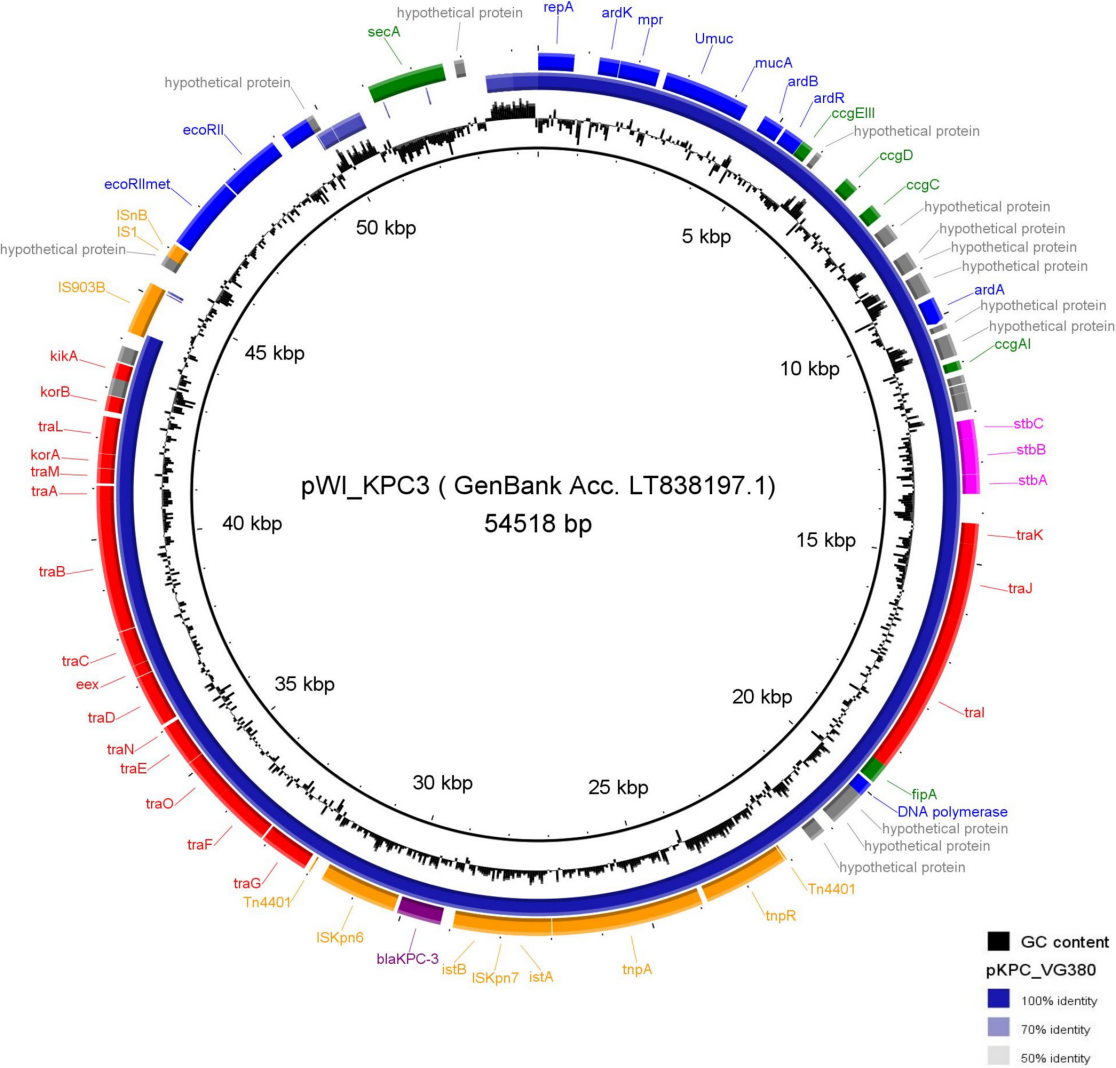
Plasmid alignment comparison between *de novo* assembled contig pKPC_VG380 (GenBank accession number PRJNA808048) found on the strain KPC-3-producing Klebsiella pneumoniae taken from a dog’s with an upper respiratory tract infection (in blue) and plasmid pWI_KPC3 (GenBank accession number LT838197.1), used as backbone plasmid reference, previously described on a French nosocomial isolate (11). Genes are represented by colored blocks: purple, resistance gene; blue, DNA replication, regulation, and restriction systems; red, conjugation-association genes; fuchsia, genes associated with partition and stability systems; orange, transposons, insertion sequences (IS), and transposase genes; green, other genes; gray, hypothetical proteins. Image generated using BRIG 0.95, available at http://brig.sourceforge.net/.

The *bla*_KPC-3_ gene was located within transposon Tn*4401d* (10) on an ~50-kb IncN-type plasmid (pKPC_VG380, pMLST ST15) (GenBank accession number PRJNA808048) (Fig. 1). When using a previously described IncN plasmid carrying the *bla*_KPC-3_ gene in Tn*4401* (pWI_KPC3, pMLST ST15) of clinical human origin (11) as a reference for BLAST, it was possible to demonstrate that one of the contigs for the KPC-3-producing K. pneumoniae ST392 VG380 had a large extent of homology with the reference plasmid (Fig. 1).

We report here, to the best of our knowledge, the first characterization of a KPC-3-producing K. pneumoniae strain, belonging to the human high-risk clonal group 147 isolated from a dog in Europe. Noteworthy, isolates belonging to the high-risk emerging lineage ST392 from CG147 and carrying the *bla*_KPC-3_ gene have been reported in a single Portuguese central hospital during 2020 (4).

To prevent spreading of carbapenem resistance, a one-health surveillance approach is urgent. The study of the epidemiology of plasmid-mediated carbapenem resistance seems to be relevant for the identification of possible pathways for antimicrobial resistance transmission.
